# Microencapsulation of *Lactobacillus plantarum* MB001 and its probiotic effect on growth performance, cecal microbiome and gut integrity of broiler chickens in a tropical climate

**DOI:** 10.5713/ab.22.0426

**Published:** 2023-05-02

**Authors:** Sasi Vimon, Kris Angkanaporn, Chackrit Nuengjamnong

**Affiliations:** 1 Department of Animal Husbandry, Faculty of Veterinary Science, Chulalongkorn University, Bangkok 10330, Thailand; 2 Department of Veterinary Physiology, Faculty of Veterinary Science, Chulalongkorn University, Bangkok 10330, Thailand; 3 Center of Excellence for Food and Water Risk Analysis (FAWRA), Faculty of Veterinary Science, Chulalongkorn University, Bangkok 10330, Thailand

**Keywords:** Broiler Chicken, Cecal Microbiome, Gut Morphology, *Lactobacillus plantarum* MB001, Microencapsulation, Nutrient Digestibility

## Abstract

**Objective:**

Microencapsulation technologies have been developed and successfully applied to protect the probiotic bacterial cells damaged by environmental exposure. This study aimed to investigate the effects of microencapsulation of *Lactobacillus plantarum* MB001 on the growth performance, ileal nutrient digestibility, jejunal histomorphology and cecal microbiome of broiler chickens in a tropical climate.

**Methods:**

A total of 288 one-day-old female broilers (Ross 308) were randomly allocated into 4 groups (6 replicates of 12 birds). Treatments included, i) a basal diet (NC), ii) NC + avilamycin (10 mg/kg) (PC), iii) NC + non-encapsulated *L. plantarum* MB001 (1×10^8^ colony-forming unit [CFU]/kg of diet) (N-LP), iv) NC + microencapsulated *L. plantarum* MB001 (1×10^8^ CFU/kg of diet) (ME-LP).

**Results:**

Dietary supplementation of ME-LP improved average daily gain, and feed conversion ratio of broilers throughout the 42-d trial period (p<0.05), whereas ME-LP did not affect average daily feed intake compared with NC group. Both N-LP and ME-LP improved apparent ileal digestibility of crude protein and ether extract compared with NC group (p<0.05). The broilers fed ME-LP supplemented diet exhibited a beneficial effect on jejunal histomorphology of villus height (VH), crypt depth (CD) and villus height to crypt depth ratio (VH:CD) of broilers compared to NC group (p<0.05). At the phylum level, Firmicutes was enriched (p<0.05) and Proteobacteria was decreased (p<0.05) only in the ME-LP group. At the genus level, the ME-LP diets increased (p<0.05) the number of both *Lactobacillus* and *Enterococcus* compared to NC, PC, and N-LP groups (p<0.05).

**Conclusion:**

Microencapsulation assists the efficient functioning of probiotics. ME-LP could be potentially used as a feed additive for improvement of cecal microbiota, gut integrity and nutrient utilization, leading to better performance of broilers.

## INTRODUCTION

Heat stress causes economic losses in the poultry industry [1] based on its negative impact on viability, immunity and growth performance in broiler chickens [2]. In this situation, antibiotics are widely used at sub-therapeutic levels as growth promoters to reduce stress and infectious diseases [3]. However, excessive and prolonged use of antibiotics in animal feeds has raised concerns regarding antibiotic residues in animal products and the development of antibiotic-resistant bacteria [4]. This has led to the banning of antibiotic use in animals in several countries [5]. The use of probiotic bacteria against pathogenic bacterial species is attracting more attention as an alternative therapy [6]. Probiotics obtained from *Lactobacillus plantarum* exhibit inhibitory action on various pathogenic bacteria, including *Listeria monocytogenes*, *Salmonella typhimurium*, *Escherichia coli* and vancomycin-resistant *Enterococci* [7]. The presence of antimicrobial metabolites, such as organic acids and bacteriocins, in *L. plantarum* can reduce the gut pH and inhibit the proliferation of opportunistic pathogens in the feed and gut of animals [8]. However, these probiotic additives are comparatively less efficient because adverse conditions including humidity, temperature and pressure during the pelleting process can lead to decreased activity [9]. Furthermore, many reports have indicated that there is poor survival of bacteria in products containing free probiotic cells during passage through the upper gastrointestinal system [10].

Several approaches that increase the resistance of these sensitive microorganisms to adverse environmental conditions have been reported. Microencapsulation has been suggested as one of the effective approaches [11]. This technique allows probiotics to survive in various unsuitable conditions and become effective into the target. The encapsulation of probiotic bacteria via microemulsion technique does not use organic solvents and is performed at room temperature. Therefore, it is a favorable way of delivering viable probiotic cells to the small intestine [12].

Sodium alginate (AL) and agar (AG) have been increasingly used to encapsulate and stabilize probiotic for intestinal delivery system due to their biocompatibility, biodegradability and non-toxicity [13]. The AL and AG are resistant to high temperature, acidic gastric juice and intestinal juice [14]. The combination of AL and AG is a good model due to potential formation of the polymer complexes [15]. However, there have been no in-depth studies of *L. plantarum* MB001 in microencapsulated form in broilers. Furthermore, no comparison between the efficacy of microencapsulated *L. plantarum* MB001 and its free cells has been ever reported.

In the present work, the novel polymer complex microencapsulation was used in manufacturing of microencapsulated *L. plantarum* MB001 (ME-LP) which comprised AL and AG as the matrix. Previously, an AL-AG matrix exhibited an effective delivery in a simulated intestinal model (Supplementary Figure S1). Thus, this study was conducted to evaluate the effects of ME-LP supplementation on growth performance, ileal nutrient digestibility, jejunal histomorphology, and cecal microbiome of broiler chickens raised under high temperature environment.

## MATERIALS AND METHODS

### Animal care

The experimental protocol was approved by the Animal Care and Ethic Committee of Faculty of Veterinary Science, Chulalongkorn University (Protocol Review No. 1931092).

### Bacterial strain and culture condition

LPMB001 was obtained from K.M.P. Biotech Co. Ltd. (Chon Buri, Thailand). The bacteria were cultivated in MRS broth and incubated at 37°C for 48 h under aerobic conditions. The cells were harvested by centrifugation at 1,500 g for 5 min at 25°C and washed twice with distilled water. Bacterial cells were declared to be 1×10^8^ viable colony-forming unit [CFU]/kg.

### Microencapsulated LPMB001

The AL (2 g) and AG (0.2 g) were dissolved in deionized water (DI) (100 mL), followed by adding tween 80 and stirring for 1 h, at 90°C to obtain AG-AL solution. AG-AL solution was maintained under constant stirring for 20 min followed by adding LPMB001 (20 mL) and soybean oil (20 mL). The solution was dropwise through a needle (diameter 1.8 mm) into CaCl_2_ (0.1 mol/L) aqueous solution to obtain microcapsules. The distance between the syringe and CaCl_2_ solution was 5 cm. Finally, the LPMB001 encapsulated AG-AL solution, namely LPMB001/AG-AL were sieved by a mesh (diameter 0.053 mm) and washed with DI for three times before drying in a hot air oven at 30°C overnight. A schematic diagram of LPMB001/AG-AL preparation is shown in Supplementary Figure S2.

### Animals, diets and management

A total of 288 one-day-old female broilers (Ross 308, initial weight 42.0±0.5 g) were provided by a local hatchery. All chicks were randomly allocated into 4 groups (6 replicates of 12 birds) including i) basal diet without additives as a negative control (NC), ii) basal diet with avilamycin at 10 ppm as a positive control (PC), iii) basal diet with free LPMB001 at the levels of 1.0×10^8^ (N-LP), and iv) basal diet with AL-AG-LPMB001 at the levels of 1.0×10^8^ (ME-LP), respectively. A feeding program consisted of 3 phases: a crumbled starter diet (days 1 to 21), a pelleted grower diet (days 22 to 35) and a pelleted finisher diet (days 36 to 42), respectively. The basal diets were formulated with corn and soybean meal to meet or exceed the nutrient requirements for Ross 308 broiler chickens [16]. All diets were pelleted at a steam conditioning temperature of 80°C for 40 s. The diet and clean tap water were provided *ad libitum* throughout the experiment. The feed ingredients and chemical compositions of the diets are shown in Table 1. The broiler chickens were reared on floor pens (100×150 cm) in a conventional house at the student training center, Department of Animal husbandry, Faculty of Veterinary Science, Chulalongkorn University, Nakhon Pathom, Thailand. A lighting program was 22 h light during the first 14 days under the incandescent lamp and then 12 h light and 12 h darkness from d 15 to the end of the trial. At 0 to 21 days (starter phase), the indoor temperature ranged from 26°C to 28°C and 32°C to 35°C in the morning and the afternoon, respectively. Relative humidity (%, RH) was recorded in the range of 83% to 85%. At 22 to 35 days (grower phase), the indoor temperature ranged from 27°C to 28°C and 33°C to 36°C in the morning and the afternoon, respectively. The RH was recorded in the range of 82% to 86%. At 36 to 42 days (finisher phase), the indoor temperature ranged from 27°C to 29°C and 34°C to 36°C in the morning and the afternoon, respectively. The RH was recorded in the range of 83% to 86%.

### Growth performance

Growth performance was measured for days 1 to 21, 22 to 42, and 1 to 42. Body weight (BW) and feed intake (FI) were recorded to calculate average daily feed intake (ADFI), and average daily gain (ADG). The feed conversion ratio (FCR) was also calculated by dividing ADFI with respective ADG. Mortality rates were recorded and calculated throughout the experiment. The weights of dead and culled birds were used to adjust for calculation of related growth performance parameters.

### Apparent ileal digestibility

The broiler chickens (3 chicks from each replicate, 18 chicks per treatment) were randomly selected, tagged using plastic band and provided 20 g/kg diet of acid-insoluble ash (AIA) as an indigestible marker in two collection periods (days 18 to 21 and days 39 to 42). At the end of two collection periods, these birds were euthanized using CO_2_ inhalation. Then, ileal digesta samples (2 to 3 g) were collected and pooled for each replicate pen. Diets and ileal contents were dried at 60°C to determine dry matter (DM), crude protein (CP), ether extract (EE), crude fiber (CF), ash, and nitrogen free extract, according to the association of official analytical chemists [17]. Gross energy was analyzed using a bomb calorimeter with benzoic acid as a reference standard (Mode AC-500; Leco, St. Joseph, MI, USA). AIA was also determined using the method of Angkanaporn et al [18]. The coefficient of apparent ileal digestibility (CAID) was calculated using the following Eq. 1.


Eq.1 $$\text{CAID}=1-[({\text{Nutrient}}_{\text{ileal digesta}}/{\text{AIA}}_{\text{ileal digesta}})∸({\text{Nutrient}}_{\text{diet}}/{\text{AIA}}_{\text{diet}})]$$

Where AIA_diet_ is the AIA concentration in the diet, AIA_digesta_ is the AIA concentration in the ileal digesta, Nutrient_digesta_ is the nutrient concentrations in the ileal digesta and Nutrient_diet_ is the nutrient concentration in the diet.

### Jejunal histomorphology

The segment of mid-jejunum (~2 to 3 cm) was taken from euthanized 3 birds (the same birds as being used for ileal digestibility) per replicate on days 21 and 42. The whole jejunum was located between endpoint of the duodenal loop and the Meckel’s diverticulum. The intestinal tissue was flushed and immediately fixed in 10% formaldehyde solution, followed by tacking in paraffin wax [19]. Afterward, fixed intestinal samples were placed on a glass slide and then stained using hematoxylin and eosin. Villus height (VH) and crypt depth (CD) were measured using a light microscope at 100× magnification (Mode BX5; Olympus, Tokyo, Japan). These data were calculated for the villus height to crypt depth (VH:CD) ratio.

### Cecal microbiome

A total of twenty-four samples of cecal content (6 from each treatment group) were randomly collected on days 21 and 42 respectively. Samples were collected in sterile containers, packed carefully, and then frozen by immersion in liquid nitrogen and stored at −80°C [19]. The cecal microbiota was analyzed using high-throughput sequencing procedure by Omics sciences and bioinformatics center of Chulalongkorn University (Bangkok, Thailand). Briefly, the procedure included DNA extraction, polymerase chain reaction (PCR)- amplified 16S rRNA, amplicon sequencing, and sequence data processing. Microbial genomic DNA was extracted from the cecal contents using a Power Soil DNA Isolation Kit (Mobio Laboratories Inc., Carlsbad, CA, USA) following the manufacturer’s recommendation. Using barcoded fusion primers, PCR amplified the V3 hypervariable region of the 16S rRNA gene from the microbial genomic DNA that was harvested from the cecal contents. The primers used were 5*′*- CCTACGGGAGGCAGCAG-3*′* with adapter A (forward primer) and 5*′* -ATTACCGCGGCTGCTGG-3*′* with adapter B (reverse primer). The PCR conditions were 94°C for 5 min; 94°C for 30 s, 48°C for 30 s, and 72°C for 30 s, repeated for 25 cycles; and 72°C for 10 min. The PCR product was excised from a 2% agarose gel and purified with a QIAGEN MinElute Gel Extraction Kit (Qiagen, Hilden, Germany). The final sequencing library was prepared by mixing equal amounts of purified PCR products and then adding poly (A) to repair the ends. Thereafter, the amplicons were connected with sequencing adapters. Following agarose gel electrophoresis, suitable fragments were selected as templates for PCR amplification. Finally, the library was sequenced by the Illumina MiSeq (San Diego, CA, USA). After sequencing, all barcodes were sorted, removed, and reads were assessed for quality. To reduce random sequencing errors, those sequences with lengths shorter than 100 bp, mismatches in PCR primers, more than one undetermined nucleotide, and an average quality of ≤25 were eliminated [19].

### Statistical analysis

All data were statistically analyzed by one-way analysis of variance using SAS statistical software [20]. Differences between mean values of the treatments were determined using post-hoc Tukey’s HSD test. The pen mean served as the experimental unit, according to the following model:


$${\text{Y}}_{\text{ij}}=\mu +{\text{T}}_{\text{i}}+{\text{e}}_{\text{ij}}$$

where Y_ij_ is the dependent variable observation, μ is the overall mean, T_i_ is the effect of the treatment, and e_ij_ is the random error. A p-value ≤0.05 was considered as a significant effect of the treatment. Results are presented as mean and standard error of mean.

## RESULTS

### Growth performance

The influence of experimental diets on growth performance of broiler chickens are summarized in Table 2. Final BW of broilers in NC was significantly lower than other groups (p< 0.01). There were no significant differences on ADFI throughout the study. During days 1 to 21, the supplementation of ME-LP at the level 1×10^8^ viable CFU/kg and PC had greater ADG (p = 0.019) than the NC group, In the finisher phase from day 22 to 42 and overall data, ADG in broilers fed with PC, N-LP, and ME-LP groups were improved compared with that in NC (p<0.01), but there were no significant differences between PC and both LP diets. Broilers fed with PC, N-LP, and ME-LP diets had lower FCR than those fed with the NC diet (p<0.05) during days 1 to 21 and improved FCR than those fed with NC diet (p = 0.034) during day 22 to 42. Overall, broilers receiving N-LP and ME-LP diets exhibited a lower FCR than those receiving NC (p = 0.029). The overall mortality rate was not affected by treatments.

### Apparent ileal digestibility

The influence of LPMB001 supplementation on CAID is shown in Table 3. On day 21, ME-LP had a significant positive effect on the CAID of CP and EE (p<0.05) compared to NC. In addition, broilers fed with ME-LP had better CAID of CP and EE than those fed with N-LP (p<0.05). However, there were no significant differences between PC and both LP diets. The CAID of DM and EE was increased by PC and ME-LP diets than that of NC diet (p<0.05) on day 42. It was noted that there were no significant differences among diets supplemented with additives.

### Jejunal histomorphology

The morphological changes of the mid-jejunal mucosa in broilers are presented in Table 4. Jejunal VH was improved by both LP treatments in comparison to NC and PC treatments on days 21 and 42 (p<0.01). However, there was no statistical difference between N-LP and ME-LP diets. On day 21, broilers fed with PC and ME-LP diets had shallower CD values than those fed with NC and N-LP diets (p<0.01). While, PC, N-LP, and ME-LP groups had lower CD than NC group (p<0.01) except that there were no significant differences between PC and ME-LP treatments as well as between N-LP and ME-LP treatments on day 42. The ME-LP diet increased VH:CD of jejunum compared with the NC and N-LP diets (p<0.01) on days 21 and 42. However, VH:CD ratio in N-LP group was greater compared with the control group (p<0.01) on days 21 and 42.

### Cecal microbiome

The 24 samples collected on day 21 and day 42 were analyzed a total of 1,541,502 and 1,368,304 of the V3 16S rRNA amplicon sequence reads respectively. The richness and diversity of cecal microflora were expressed in terms of the number of operational taxonomic units (OTUs). However, no significant differences of OTUs were found among all groups on days 21 and 42. The Chao1 and Shannon indices of cecal microflora showed no difference between any groups (Table 5). The taxon abundance of each sample was further analyzed at the level of phylum and genus using mainly the database of the Ribosomal Database Project with a bootstrap confidence threshold of 80%. In both phases, the dominant phylum was Firmicutes, which comprised 68.44% to 72.67% of the samples on day 21, and 69.98% to 85.67% of the samples on day 42. On day 42, there were a number of Firmicutes that were enriched (p<0.05) in the ME-LP group compared with the NC, PC and N-LP groups, whereas the Proteobacteria were decreased (p<0.05) in the ME-LP group compared with the NC, PC, and N-LP groups as well. Furthermore, no differences were found in either the Bacteroidetes or the Tenericutes on days 21 and 42 (Table 6). At the genus level, the ME-LP diets increased (p<0.05) the number of *Lactobacillus* and *Enterococcus* in both the starter and finisher phases. No differences in quantities were found in other bacterial genera among any of the groups in either phase (Table 7).

## DISCUSSION

This experiment was conducted in a conventional broiler house during the summer period. Broilers raised in environment with high temperature (above 30°C for 10 hours per day for consecutive 20 days) frequently experienced chronic HS, leading to lower growth performance [1]. In this research, it was investigated if broilers may build a resistance to chronic heat stress through the alteration of natural gut microflora via microencapsulation. In a wide range of industries, microencapsulation has been recognized as a viable method for enhancing additive performance. This method has the potential to enhance the release qualities, extend shelf life, cover undesirable tastes as well as improve thermostability. Rosenberg et al [21] reported that microencapsulation technology increases the survival of the probiotic bacteria to as much as 80% to 95%. It was revealed that performance of broilers in this trial showed lower FI and BW than the standard Ross 308 broiler under conditions of high ambient temperature [22]. However, N-LP was effective in enhancing ADG and FCR compared to NC in the overall period. It has been shown in previous research with heat-stressed chickens that birds fed on dietary supplements containing *Lactobacillus* strains showed significant improvements in ADG and FI [23]. Moreover, there were significant improvements of BW, weight gain, FI, and FCR of broilers fed on *Lactobacillus* strains compared with broilers fed oxytetracycline at a sub-therapeutic dose under heat stress [24]. Probiotics have been demonstrated in numerous studies to enhance nutrient absorption, which in turn enhances the growth performance of broilers under heat stress. Jahromi et al [25] reported that probiotics promoted broilers’ growth performance, which may result from the enhancement of nutrient transporter gene expression (Na+-dependent glucose [*SGLNC*], galactose transporter [*SGLRI11*] and long-chain acyl CoA dehydrogenase genes) under heat stress. Similarly, Navidshad et al [26] showed that increased utilization of nutrients as a result upregulation of nutrient gene expression leads to improvement of the BW of broilers. In contrast, several studies did not report beneficial effects of probiotics on the growth performance of broiler chickens [27,28], This inconsistency might be attributed to the strains of the probiotics, administration dosage or the forms of the probiotics. In the current experiment, *L. plantarum* MB001, including free non-encapsulated (N-LP) and microencapsulated (ME-LP) forms, was added into the diet at 1×10^8^ CFU/kg in broiler chickens. Our data showed that the addition of the ME-LP demonstrated a significant increase in ADG. Moreover, the ME-LP group decreased the FCR significantly when compared with the NC and N-LP. groups. In agreement with our results, Gbassi et al [9] reported that broilers could improve ADG and FCR when the basal diet was supplemented with *Lactobacillus plantarum* microcapsule. These results indicate that the ME-LP potentially exhibits similar effects to antibiotics on the growth performance and dietary manipulation with ME-LP might ameliorate the adverse impacts on the growth performance of chickens raised under tropical conditions.

In general, the small intestine is a target site for the evaluation of probiotic properties. With modulation of the gut microbiota, jejunal morphology changes in broilers are noted. These include changes in the integrity of the gut wall and the rate at which cells undergo apoptosis [21]. The VH and CD are important indicators of gut function and animal health. The villi are one of the key components responsible for the absorption of nutrients in the small intestine. Increasing of VH and decreased CD may result in higher nutrient absorption, reduced secretion in the gastrointestinal tract and improvement of growth performance [21]. The result indicated that broilers in NC group had the shortest VH, deepest CD and the lowest VH:CD, whereas a positive impact was seen in the ME-LP group. Moreover, the ME-LP supplemented group had slightly more positive effects on VH, CD, and VH:CD of jejunum than N-LP group. This evidence indicates the vital role of ME-LP to improve intestinal morphology. Probiotic bacteria regulate epithelial permeability by modulating tight junction proteins, which in turn inhibits pathogen colonization, modulates cell proliferation and apoptosis, and controls mucin production [29]. Bacterial toxins are known to have negative effects on intestinal morphology. It has been reported that *L. plantarum* eliminates pathogenic bacteria by producing bacterial toxins called "bacitracin". [30]. As a result, bacitracin limits the production of toxic compounds and reduces damage to intestinal epithelial cells of broiler chickens. Consistent with our results, Awad et al [31] found that inclusion of *Lactobacillus* spp. increased the length of villi and their absorptive surface areas of broilers, thus increasing BW. Moreover, the improvement of villi height and epithelial cell function, which might be the reason for enhanced nutrient uptake efficiency and improved CAID of nutrients. Likewise, the improvement of VH:CD involves the turnover rate of epithelial cells which reduced energy for maintaining the gut function and increased energy reserves [32]. Hence, our results indicate that the ME-LP were effective at promoting the intestinal histomorphology of broilers, despite the negative impact of heat stress.

The improvement in the gut integrity is associated with nutrient digestibility. In this study, the ME-LP group had higher ileal CAID of CP and EE compared with the NC group. ME-LP exerted greater CAID of CP and EE by 15% and 9% for 21 day and by 19% and 8% for day 42 in comparison to NC, respectively. The high CP digestibility in ME-LP group may enhance bioavailable amino acids and cell proliferation, resulting in increased intestinal integrity. An improved nutrient digestibility is concomitant with ME-LP supplement because these probiotics are thought to result in more nutrients becoming available for absorption via the suppression of growth and metabolic activities of the gut pathogens, as well as alterations in intestinal growth, morphology, and function such as reduction of intestinal epithelium thickness and epithelial cell turnover. Moreover, this may probably involve the stimulated secretion of bile, mucus, and endogenous enzymes (trypsin, chymotrypsin, lipase, and amylase) in the pancreas and intestinal wall by probiotic bacteria [33]. Similarly, the adding of *Lactobacillus* spp. and *Bifidobacterium* spp. encouraged apparent total tract digestibility of DM, CP, and EE in broilers orally challenged with *Clostridium perfringens* [29]. In this study, PC exhibited no significant effect on the CAID (p<0.05) compared with ME-LP. Nevertheless, probiotics represent a different concept from PC, whereby the intake of live microorganisms is aimed to modulate the gut environment and enhance the gut barrier function via the fortification of the beneficial members of the intestinal microflora, the competitive exclusion of pathogens, and the stimulation of the immune system [34]. On the other hand, PC modulate the bacterial community and enhance the CAID via the suppression of all intestinal microflora. The CAID result was positively linked to growth response.

The intestinal microbiota is a vital determinant of gastrointestinal health. A balanced microbial population was able to support a healthy intestinal tract resulting in better control of intestinal pathogens [35]. Environmental stressors disturb the stability of the intestinal microbial ecology, resulting in dysbiosis [36]. Probiotics have a beneficial effect by helping to maintain normal intestinal microbiota. The present study evaluated whether such beneficial effects of probiotics on cecal microbiota could be replicated under heat stress. The current results showed that the species composition of gut microbial communities was influenced by probiotics (N-LP and ME-LP). The Shannon diversity index significantly increased with the addition of ME-LP when compared with other groups, which was similar to the previous studies. Song et al [35] reported that supplementation with probiotics could change the diversity of bacteria in the cecum of chickens. The major bacterial phyla in the chicken-gut microbiota included Firmicutes and Proteobacteria. Recent data revealed that the gut microbiota, especially Firmicutes and Proteobacteria, could contribute to host metabolism by several mechanisms including increase of energy derived from the diet, modulation of lipid metabolism, alteration of endocrine function [37]. For overall period, the dietary inclusion of ME-LP increased the cecal Firmicutes and decreased the cecal Proteobacteria compared with those of the NC, PC, and N-LP groups (p<0.05). The results were consistent with the report that the gradual increase of Firmicutes at the expense of Proteobacteria was correlated with BW increases during chicken rearing [38]. Moreover, the ME-LP group significantly increased the cecal *Lactobacillus* and *Enterococcus* counts compared with those of the NC and PC groups. The greater survival of the *Enterococcus* and *Lactobacillus* confirmed that ME-LP offered better protection against gastrointestinal disorders. Loh et al [39] reported that increased lactic acid bacteria populations have been reported following supplementation of broiler and layer feeds with four combinations of *L. plantarum*-derived metabolites. The main reason for such an effect is that probiotics have the capacity to promote the growth of beneficial bacteria (*Lactobacillus* and *Enterococcus*) while hindering the growth of pathogenic bacteria in the gut epithelium [39]. It has been reported that *L. plantarum* contain bacteriocins, short-chain fatty acids (SCFAs), organic acids that result in the reduction of gut pH [35]. Bacterial species such as *E. coli* and *Salmonella* are intolerant to acidic environments, thus the actions of bacteriocins and SCFAs from probiotics inhibit their activities [26]. Another role of these beneficial bacteria includes competition for intestinal adhesion sites and nutrients and eliciting immune responses. As compared with those in the N-LP group, the ME-LP with AL and AG decreased the Proteobacteria counts and increased the numbers of *Lactobacillus* and *Enterococcus*, in all periods of age. Microencapsulation could function as a protective means of ensuring viability of probiotics. Similarly, AL particles sustained the survival rate of *Lactobacillus acidophilus* better than its free form [40]. Zhang et al [19] reported that probiotic microcapsules did not change the diversity and richness of the intestinal ecosystem. Nevertheless, the different outputs of microbial community may be due to variable compositions and dosages of probiotics, basal diets, animal condition, and experimental condition [41]. The viability of N-LP and ME-LP after passing through feed processing and the digestive system was described as follows. The ME-LP was partially lost in feed processing during hydrothermal processing whereas the unique characteristic of AL-AG matrix improved the stability of ME-LP. The N-LP entering the proventriculus may stimulate the secretion of hydrochloric acid (HCl) and pepsin, leading to increased digestibility. However, the movement of N-LP into the intestine was relatively limited because of its poor viability in HCl. Morphological changes of ME-LP were found in proventriculus as follows. The matrix of ME-LP (AL-AG) was swollen because of the gastric pH environment (Supplementary Figure S3). This might cause the protonation of carboxylate groups, resulting in the chain expansion and absorption of a large amount of water. In the next step, the destruction of cross-linker was disturbed due to the ion-exchange in simulated gastrointestinal fluids (SGF). The erosion of microcapsule structure facilitated the penetration of SGF into ME-LP microcapsules. However, the carboxylate groups of AG-AL chains and their networks in SGF still maintained ME-LP under pH variations and even in endogenous enzymes in the gut. In the final step, the crosslink network of AG-AL was gradually degraded as AG-AL started to deprotonate in simulated intestinal fluids (SIF) resulting in the diffusion of *L. plantarum*. According to Zhang et al [19] who reported that AL microcapsules of probiotics showed a delayed release in stomach and subsequently functioned in intestine. The release mechanism, as previously described, was dependent on type of polymer and its characteristic, cross-linking degree, environmental conditions, and incubation time. The ME-LP was initially dissolved at pH>5 due to the protonation of the ionic carboxylate groups, leading to the diffusion of *L. plantarum* into lower part of intestine. Consequently, ME-LP may maintain the most possible amount of bioactive compound, thus facilitating intestinal function. This study showed that the ME-LP significantly decreased the number of pathogenic bacteria even under heat-stress conditions. These results might help to explain why the use of the ME-LP results in improved growth performance equally the use of the avilamycin in broiler chicken feed.

## CONCLUSION

Probiotic *L. plantarum* MB001 can be one of the potential alternatives for antibiotics in broiler feed. In comparison with non-encapsulated probiotic, the microencapsulated probiotic (ME-LP) has more beneficial effect in improving FCR, ADG, VH/CD ratio and cecal microbiota of broilers reared under tropical condition.

## Figures and Tables

**Table 1:** Ingredients and calculated composition of the basal diet (as-fed basis)

Items	Feeding phase		

	Starter	Grower	Finisher
Ingredient (%)			
Corn	43.0	46.0	49.0
Cassava	6.0	8.0	10.0
Full-fat soybean	8.5	8.0	7.5
Soybean meal (44%)	32.0	28.0	24.0
Salt stone 50%	1.5	0.6	0.6
Limestone	1.3	1.2	1.1
Mono-dicalcium phosphate	1.4	1.4	1.3
Rice bran oil	4.6	5.2	5.4
Antimould agent^1)^	0.1	0.1	0.1
DL-Methionine	0.27	0.2	0.2
L-lysine HCl	0.03	0	0
Choline chloride 75%	0.2	0.2	0.1
Absorbent toxins^2)^	0.2	0.2	0.1
Premix^3)^	0.8	0.8	0.5
Sodium bicarbonate	0.1	0.1	0.1
Total	100	100	100
Calculated nutrient composition (%)			
Dry matter	87.9	87.0	87.0
Metabolizable energy (kcal/kg)	3,100	3,150	3,200
Crude protein	22.0	20.0	19.0
Ether extract	6.5	7.0	7.5
Crude fiber	3.5	4.0	4.0
Ash	5.76	5.06	5.15
Methionine	0.63	0.55	0.54
Lysine	1.44	1.29	1.16
Met+Cys	0.98	0.80	0.75
Calcium	0.9	0.85	0.8
Total phosphorus	0.78	0.70	0.68
Available phosphorus	0.45	0.40	0.37

1) Propionic acid.

2) HSCAS, hydrate sodium calcium aluminosilicate.

3) Premix provided the following per kg of diet: vitamin A 12,500 IU, vitamin D_3_ 16,000,000 IU, vitamin E 100 mg, vitamin K_3_ 4 mg, vitamin B_1_ 3 mg, vitamin B_2_ 9 mg, vitamin B_6_ 6 mg, vitamin B_12_ 0.03 mg, niacin 80 mg, pantothenic acid 18 mg, folic acid 2.5 mg, biotin 0.40 mg, vitamin C 200 mg, zinc 96.0 g, iron 64.0 g, copper 12.8 g, iodine 0.80 g, cobalt 0.16 g, selenium 0.16 g.

**Table 2:** Growth performance of broiler chickens on day 21 and day 42^1)^

Items	Dietary treatment^2)^				SEM	p-value

	NC	PC	N-LP	ME-LP		
Initial body weight (g)	42.1	42.5	41.5	41.7	0.48	0.833
Final body weight (g)	2,620^b^	2,787^a^	2,698^ab^	2,818^a^	1.20	0.005
Average daily feed intake (g/d)						
Day 1 to 21	65.7	64.5	64.6	65.0	0.50	0.296
Day 22 to 42	159.6	157.2	158.7	157.1	1.70	0.246
Day 1 to 42	122.4	117.7	119.9	119.4	1.00	0.067
Average daily gain (g/d)						
Day 1 to 21	40.2^b^	41.7^a^	41.0^ab^	42.0^a^	0.30	0.019
Day 22 to 42	82.3^c^	86.9^ab^	84.8^b^	89.9^a^	1.10	0.008
Day 1 to 42	61.2^c^	64.3^ab^	63.0^b^	65.9^a^	0.60	0.007
Feed conversion ratio						
Day 1 to 21	1.63^b^	1.55^a^	1.57^a^	1.55^a^	0.01	0.031
Day 22 to 42	1.95^c^	1.81^ab^	1.87^b^	1.76^a^	0.04	0.034
Day 1 to 42	2.00^c^	1.83^a^	1.90^b^	1.82^a^	0.02	0.029
Mortality (%)	2.0	1.0	1.0	1.0	1.00	0.753

SEM, standard error of the mean.

1) Data represent the mean of 6 replicates.

2) NC, broiler fed a basal diet without additives; PC, broiler fed a basal diet with avilamycin at 10 ppm; N-LP and ME-LP: broiler fed a basal diet with unencapsulated *L. plantarum* MB001 and microencapsulated *L. plantarum* MB001 at the level 1×10^8^ viable CFU/kg.

a–c Different superscript letters indicate significant differences in the rows (p<0.05).

**Table 3:** Coefficient of apparent ileal digestibility (CAID) of broiler chickens on day 21 and day 42^1)^

Items	Dietary treatment^2)^				SEM	p-value

	NC	PC	N-LP	ME-LP		
Day 21						
Dry matter	0.609	0.710	0.680	0.719	0.01	0.058
Crude protein	0.682^c^	0.768^ab^	0.728^b^	0.801^a^	0.77	0.025
Ether extract	0.790^c^	0.832^ab^	0.815^b^	0.866^a^	1.63	0.009
Day 42						
Dry matter	0.615^b^	0.682^a^	0.656^ab^	0.698^a^	0.01	0.003
Crude protein	0.678	0.759	0.731	0.763	0.76	0.054
Ether extract	0.792^b^	0.853^a^	0.824^ab^	0.857^a^	0.03	0.047

SEM, standard error of the mean.

1) Data represent the mean of 6 replicates.

2) NC, broiler fed a basal diet without additives; PC, broiler fed a basal diet with avilamycin at 10 ppm; N-LP and ME-LP, broiler fed a basal diet with unencapsulated *L. plantarum* MB001 and microencapsulated *L. plantarum* MB001 at the level 1×10^8^ viable CFU/kg.

a–c Different superscript letters indicate significant differences in the rows (p<0.05).

**Table 4:** Jejunal histomorphology of broiler chickens on days 21 and day 42^1)^

Items	Dietary treatment^2)^				SEM	p-value

	NC	PC	N-LP	ME-LP		
Day 21						
Villus height (VH, μm)	606.36^c^	698.57^b^	719.06^a^	720.50^a^	1.25	<0.001
Crypt depth (CD, μm)	90.01^c^	76.67^a^	85.61^b^	75.48^a^	2.23	<0.001
VH/CD ratio	6.74^c^	9.11^a^	8.40^b^	9.54^a^	0.12	<0.001
Day 42						
Villus height (VH, μm)	584.54^c^	654.19^b^	705.28^a^	716.15^a^	3.56	0.001
Crypt depth (CD, μm)	97.12^a^	77.59^c^	89.49^b^	82.59^bc^	3.45	0.001
VH/CD ratio	6.02^c^	8.43^a^	7.88^b^	8.67^a^	0.27	0.010

SEM, standard error of the mean.

1) Data represent the mean of 6 replicates.

2) NC, broiler fed a basal diet without additives; PC, broiler fed a basal diet with avilamycin at 10 ppm; N-LP and ME-LP, broiler fed a basal diet with unencapsulated *L. plantarum* MB001 and microencapsulated *L. plantarum* MB001 at the level 1×10^8^ viable CFU/kg.

a–c Different superscript letters indicate significant differences in the rows (p<0.05).

**Table 5:** Number of operational taxonomic units (OTU) per group and estimators of sequence diversity and richness in broiler chickens on days 21 and day 42^1)^

Items	Dietary treatment^2)^				SEM	p-value

	NC	PC	N-LP	ME-LP		
Day 21						
Number of operational taxonomic unit	50,730	69,976	72,564	88,956	1,097	0.879
Chao1 (richness)	10,182	9,895	11,742	12,635	737	0.877
Shannon (diversity)	6.27	5.92	6.83	6.97	0.36	0.721
Day 42						
Number of operational taxonomic unit	54,489	64,159	70,281	76,152	996	0.673
Chao1 (richness)	12,453	11,908	13,566	14,673	645	0.845
Shannon (diversity)	6.92^b^	6.56^c^	7.01^b^	7.25^a^	0.45	0.010

SEM, standard error of the mean.

1) Data represent the mean of 6 replicates.

2) NC, broiler fed a basal diet without additives; PC, broiler fed a basal diet with avilamycin at 10 ppm; N-LP and ME-LP, broiler fed a basal diet with unencapsulated *L. plantarum* MB001 and microencapsulated *L. plantarum* MB001 at the level 1×10^8^ viable CFU/kg.

a–c Different superscript letters indicate significant differences in the rows (p<0.05).

**Table 6:** Bacterial phyla distributions of broiler chickens on days 21 and day 42^1)^

Phyla (%)	Dietary treatment^2)^				SEM	p-value

	NC	PC	N-LP	ME-LP		
Day 21						
Firmicutes	68.44	70.43	69.28	72.67	2.42	0.509
Bacteroidetes	19.90	18.45	20.67	21.23	1.27	0.482
Proteobacteria	6.33	5.11	4.02	3.67	0.77	0.945
Tenericutes	1.71	0.92	1.53	1.14	0.05	0.422
Day 42						
Firmicutes	69.98^c^	70.93^c^	77.58^b^	85.67^a^	0.98	0.005
Bacteroidetes	12.05	13.85	15.89	16.93	3.45	0.085
Proteobacteria	4.72^c^	4.11^c^	3.03^b^	1.69^a^	0.40	0.003
Tenericutes	0.93	0.62	1.16	0.99	0.05	0.420

SEM, standard error of the mean.

1) Data represent the mean of 6 replicates.

2) NC, broiler fed a basal diet without additives; PC, broiler fed a basal diet with avilamycin at 10 ppm; N-LP and ME-LP, broiler fed a basal diet with unencapsulated *L. plantarum* MB001 and microencapsulated *L. plantarum* MB001 at the level 1×10^8^ viable CFU/kg.

a–c Different superscript letters indicate significant differences in the rows (p<0.05).

**Table 7:** Bacterial genera distribution of broiler chickens on days 21 and day 42^1)^

Genus (%)	Dietary treatment^2)^				SEM	p-value

	NC	PC	N-LP	ME-LP		
Day 21						
*Faecalibacterium*	1.32	1.67	1.60	2.14	0.65	0.384
*Ruminococcus*	2.80	3.57	5.25	6.60	0.57	0.725
*Oscil lospira*	5.84	5.24	6.22	4.59	0.23	0.793
*Coprococcus*	1.21	1.14	0.95	0.71	0.01	0.195
*Clostridium*	0.16	0.19	0.20	0.22	0.02	0.541
*Lactobacillus*	0.57^c^	0.42^c^	0.73^b^	0.82^a^	<0.01	<0.001
*Enterococcus*	0.09^c^	0.07^c^	0.14^b^	0.20^a^	<0.01	<0.001
Day 42						
*Faecalibacterium*	14.81	12.66	15.45	17.42	3.70	0.589
*Ruminococcus*	5.64	3.92	5.25	5.76	0.63	0.516
*Oscil lospira*	5.15	3.46	5.87	5.99	0.27	0.808
*Coprococcus*	0.34	0.38	0.37	0.37	0.06	0.509
*Clostridium*	1.41	1.43	0.55	0.33	0.05	0.299
*Lactobacillus*	0.51^c^	0.38^c^	0.70^b^	0.88^a^	<0.01	<0.001
*Enterococcus*	0.06^c^	0.05^c^	0.15^b^	0.23^a^	<0.01	<0.001

SEM, standard error of the mean.

1) Data represent the mean of 6 replicates.

2) NC, broiler fed a basal diet without additives; PC, broiler fed a basal diet with avilamycin at 10 ppm; N-LP and ME-LP, broiler fed a basal diet with unencapsulated *L. plantarum* MB001 and microencapsulated *L. plantarum* MB001 at the level 1×10^8^ viable CFU/kg.

a–c Different superscript letters indicate significant differences in the rows (p<0.05).

## SUPPLEMENTARY MATERIAL

**Supplementary Figure S1.** The thermogravimetric analysis (TGA) thermograms of (A) LPMB001, (B) AL, (C) AG, and (D) LPMB001/AG-AL.

**Supplementary Figure S2.** Schematic for steps of preparation of LPMB001/AG-AL.

**Supplementary Figure S3.** Performances of LPMB001/AG-AL in terms of (A) swelling percent (%), and (B) cumulative release (%) in SGF and SIF at 39.5°C±0.5°C.

## References

[1] Attia YA, Al-Harthi MA, Elnaggar A (2018). Productive, physiological and immunological responses of two broiler strains fed different dietary regimens and exposed to heat stress. Ital J Anim Sci.

[2] Celi P, Verlhac V, Pérez Calvo E, Schmeisser J, Kluenter AM (2019). Biomarkers of gastrointestinal functionality in animal nutrition and health. Anim Feed Sci Technol.

[3] Lara LJ, Rostagno MH (2013). Impact of heat stress on poultry production. Animal.

[4] Van den Bogaard AE, Stobberingh EE (2000). Epidemiology of resistance to antibiotics: links between animals and humans. Int J Antimicrob Agents.

[5] De Vos P, Faas MM, Spasojevic M, Sikkema J (2010). Encapsulation for preservation of functionality and targeted delivery of bioactive food components. Int Dairy J.

[6] Mountzouris KC, Tsitrsikos P, Palamidi I (2010). Effects of probiotic inclusion levels in broiler nutrition on growth performance, nutrient digestibility, plasma immunoglobulins, and cecal microflora composition. Poult Sci.

[7] Chen H, Li X, Liu B, Meng X (2017). Microencapsulation of Lactobacillus bulgaricus and survival assays under simulated gastrointestinal conditions. J Funct Foods.

[8] Etchepare MA, Raddatz GC, Flores EM (2016). Effect of resistant starch and chitosan on survival of Lactobacillus acidophilus microencapsulated with sodium alginate. LWT – Food Sci Technol.

[9] Gbassi GK, Vandamme T, Yolou FS, Marchioni E (2011). In vitro effects of pH, bile salts and enzymes on the release and viability of encapsulated Lactobacillus plantarum strains in a gastrointestinal tract model. Int Dairy J.

[10] Gebara C, Chaves KS, Ribeiro MCE, Souza FN, Grosso CRF, Gigante ML (2013). Viability of Lactobacillus acidophilus La5 in pectin–whey protein microparticles during exposure to simulated gastrointestinal conditions. Food Res Int.

[11] Han W, Zhang XL, Wang DW (2013). Effects of microencapsulated Enterococcus fecalis CG1.0007 on growth performance, antioxidation activity, and intestinal microbiota in broiler chickens. J Anim Sci.

[12] Annan NT, Borza AD, Hansen LT (2008). Encapsulation in alginate-coated gelatin microspheres improves survival of the probiotic Bifidobacterium adolescentis 15703T during exposure to simulated gastro-intestinal conditions. Food Res Int.

[13] Dajian H, Qiling Q, Yuting Z, Wenjie T, Zhuo Z, Xiaohu Q (2020). Dual-network design to enhance the properties of agar aerogel adsorbent by incorporating in situ ion cross-linked alginate. Environ Chem Lett.

[14] Xiubin H, Zhixin X, Yanzhi X (2019). Effect of SiO2 nanoparticle on the physical and chemical properties of eco-friendly agar/sodium alginate nanocomposite film. Int J Biol Macromol.

[15] Krasaekoopt W, Watcharapoka S (2014). Effect of addition of inulin and galactooligosaccharide on the survival of microencapsulated probiotics in alginate beads coated with chitosan in simulated digestive system, yogurt and fruit juice. LWT – Food Sci Technol.

[16] Aviagen (2018). Ross broiler management manual.

[17] AOAC International (2016). Official methods of analysis of association of official analytical chemists International.

[18] Angkanaporn K, Ravindran V, Bryden WL (1996). Additivity of apparent and true ileal amino acid digestibilities in soybean meal, sunflower meal, and meat and bone meal for broilers. Poult Sci.

[19] Zhang L, Li J, Yun TT (2015). Effects of pre-encapsulated and pro-encapsulated Enterococcus faecalis on growth performance, blood characteristics, and cecal microflora in broiler chickens. Poult Sci.

[20] SAS Institute (1985). SAS user’s guide: Statistics, version.

[21] Rosenberg M, Lee SJ (2004). Calcium-alginate coated, whey protein-based microspheres: Preparation, some properties and opportunities. J Microencapsul.

[22] Rather SA, Akhter R, Masoodi FA, Gani A, Wani SM (2017). Effect of double alginate microencapsulation on in vitro digestibility and thermal tolerance of Lactobacillus plantarum NCDC201 and L. casei NCDC297. Lebensmittel-Wissenschaft and Technologie Food Sci Technol.

[23] Jahromi MF, Liang JB, Ebrahimi R (2017). Protective potential of Lactobacillus species in lead toxicity model in broiler chickens. Animal.

[24] Zulkifli I, Abdullah N, Azrin NM, Ho YW (2000). Growth performance and immune response of two commercial broiler strains fed diets containing Lactobacillus cultures and oxytetracycline under heat stress conditions. Br Poult Sci.

[25] Jahromi MF, Altaher YW, Shokryazdan P (2016). Dietary supplementation of a mixture of Lactobacillus strains enhances performance of broiler chickens raised under heat stress conditions. Int J Biometeorol.

[26] Navidshad B, Liang JB, Jahromi MF (2012). Correlation coefficients between different methods of expressing bacterial quantification using real time PCR. Int J Mol Sci.

[27] Lee KW, Lee SH, Lillehoj HS (2010). Effects of direct-fed microbials on growth performance, gut morphometry, and immune characteristics in broiler chickens. Poult Sci.

[28] Li LL, Hou ZP, Li TJ (2008). Effects of dietary probiotic supplementation on ileal digestibility of nutrients and growth performance in 1- to 42-day-old broilers. J Sci Food Agric.

[29] Zeng Z, Xu X, Zhang Q (2015). Effects of essential oil supplementation of a low-energy diet on performance, intestinal morphology and microflora, immune properties and antioxidant activities in weaned pigs. Anim Sci J.

[30] Thu TV, Loh TC, Foo HL, Yaakub H, Bejo MH (2011). Effects of liquid metabolite combinations produced by Lactobacillus plantarum on growth performance, faeces characteristics, intestinal morphology and diarrhea incidence in postweaning piglets. Trop Anim Health Prod.

[31] Awad WA, Böhm J, Razzazi-Fazeli E, Ghareeb K, Zentek J (2006). Effect of addition of a probiotic microorganism to broiler diets contaminated with deoxynivalenol on performance and histological alterations of intestinal villi of broiler chickens. Poult Sci.

[32] Kareem KY, Loh TC, Foo HL, Akit H, Samsudin AA (2016). Effects of dietary postbiotic and inulin on growth performance, IGF1 and GHR mRNA expression, faecal microbiota and volatile fatty acids in broilers. BMC Vet Res.

[33] Obajuluwa OV, Sanwo KA, Egbeyale LT, Fafiolu AO (2020). Performance, blood profile and gut morphometry of broiler chickens fed diets supplemented with Yohimbe (Pausynistalia yohimbe) and Larvacide. Vet Anim Sci.

[34] Vicente JL, Torres-Rodriguez A, Higgins SE (2008). Effect of a selected Lactobacillus spp.-based probiotic on Salmonella enterica serovar enteritidis-infected broiler chicks. Avian Dis.

[35] Song D, Wang YW, Lu ZX (2019). Effects of dietary supplementation of microencapsulated Enterococcus fecalis and the extract of Camellia oleifera seed on laying performance, egg quality, serum biochemical parameters, and cecal microflora diversity in laying hens. Poult Sci.

[36] Suzuki K, Harasawa R, Yoshitake Y, Mitsuoka T (1983). Effects of crowding and heat stress on intestinal flora, body weight gain, and feed efficiency of growing rats and chicks. Nihon Juigaku Zasshi.

[37] Greiner T, Bäckhed F (2011). Effects of the gut microbiota on obesity and glucose homeostasis. Trends Endocrinol Metab.

[38] Videnska P, Sedlar K, Lukac M (2014). Succession and replacement of bacterial populations in the caecum of egg laying hens over their whole life. PLoS One.

[39] Loh T, Thanh N, Foo H, Hair-Bejo M (2013). Effects of feeding metabolite combinations from Lactobacillus plantarum on plasma and breast meat lipids in broiler chickens. Rev Bras Cienc Avic.

[40] Marteau P, Shanahan F (2003). Basic aspects and pharmacology of probiotics: An overview of pharmacokinetics, mechanisms of action and side-effects. Best Pract Res Clin Gastroenterol.

[41] Rimoldi S, Lasagna E, Sarti FM (2015). Expression profile of six stress-related genes and productive performances of fast and slow growing broiler strains reared under heat stress conditions. Meta Gene.

