# Supplementary material for: Microencapsulation of Lactobacillus plantarum MB001 and its probiotic effect on growth performance, cecal microbiome and gut integrity of broiler chickens in a tropical climate
Source: Anim Biosci. 2023 May 2;36(8):1252–62. doi: 10.5713/ab.22.0426 (PMC10330975; doi:10.5713/ab.22.0426)
Supplement: Supplementary file 1 [file ab-22-0426-Supplementary-Fig-1.pdf]

SUPPLEMENTARY MATERIALS

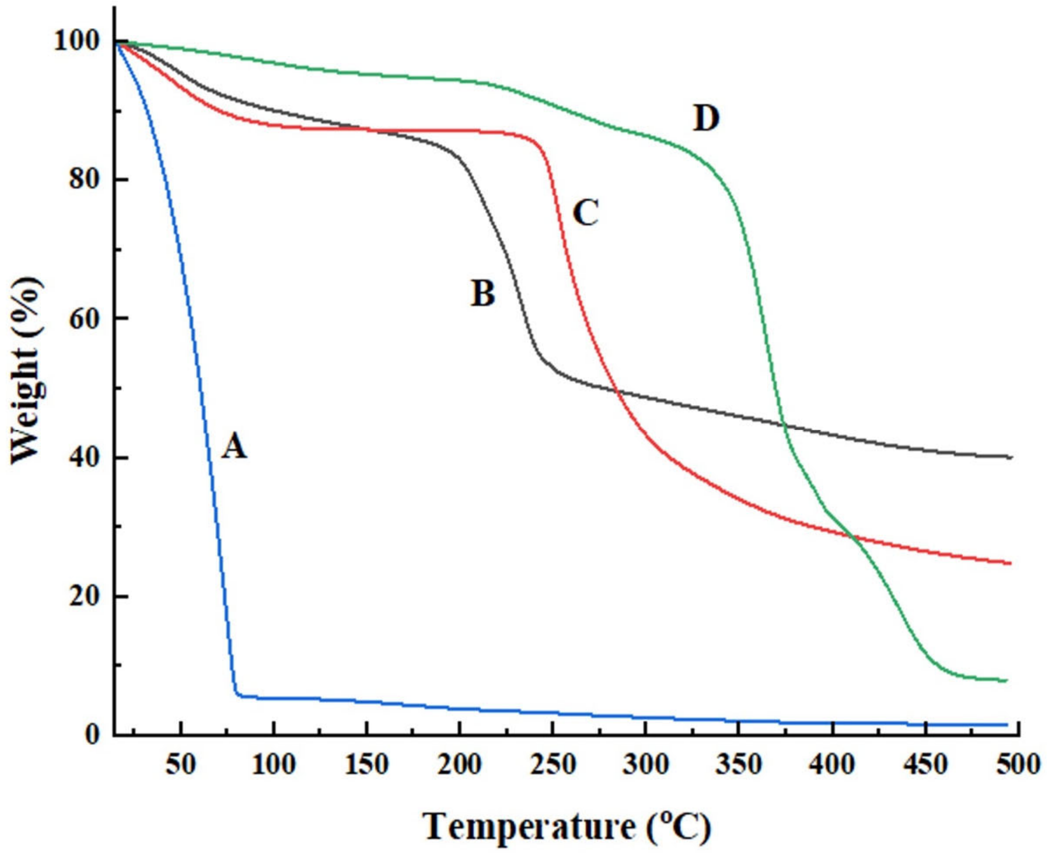

Figure S1. The thermogravimetric analysis (TGA) thermograms of (A) LPMB001, (B) AL, (C) AG and (D) LPMB001/AG-AL.
