# Supplementary figures and images for: Microencapsulation of Lactobacillus plantarum MB001 and its probiotic effect on growth performance, cecal microbiome and gut integrity of broiler chickens in a tropical climate
Source: Anim Biosci. 2023 May 2;36(8):1252–62. doi: 10.5713/ab.22.0426 (PMC10330975; doi:10.5713/ab.22.0426)

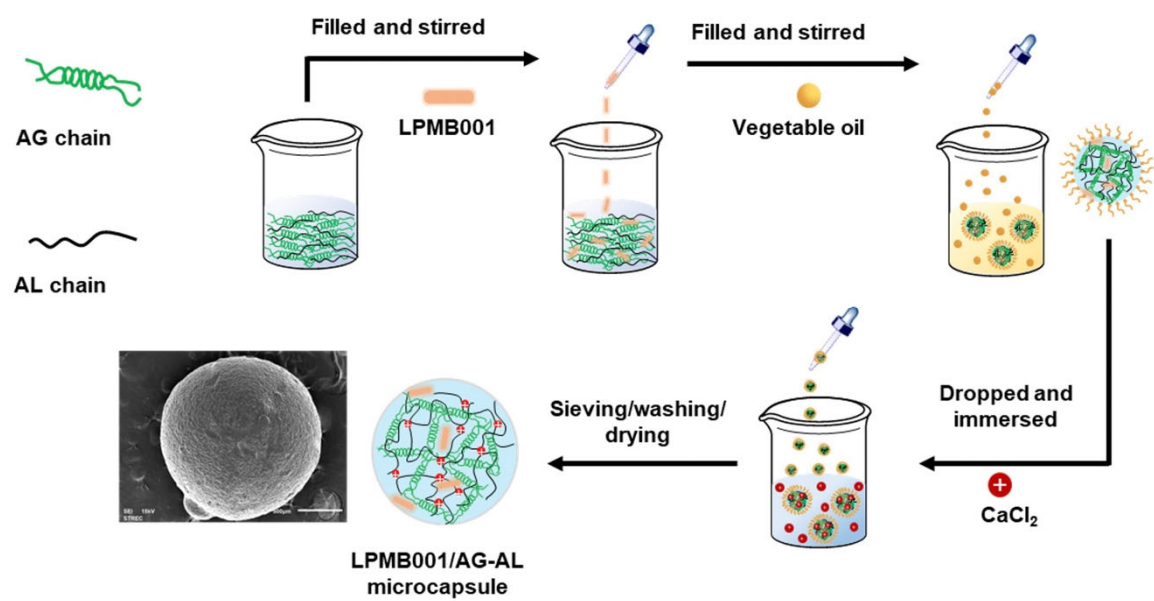

**Figure S2.** Schematic for steps of preparation of LPMB001/AG-AL.

Supplement: Supplementary file 2 [file ab-22-0426-Supplementary-Fig-2.pdf]
