# Supplementary material for: Microencapsulation of Lactobacillus plantarum MB001 and its probiotic effect on growth performance, cecal microbiome and gut integrity of broiler chickens in a tropical climate
Source: Anim Biosci. 2023 May 2;36(8):1252–62. doi: 10.5713/ab.22.0426 (PMC10330975; doi:10.5713/ab.22.0426)
Supplement: Supplementary file 3 [file ab-22-0426-Supplementary-Fig-3.pdf]

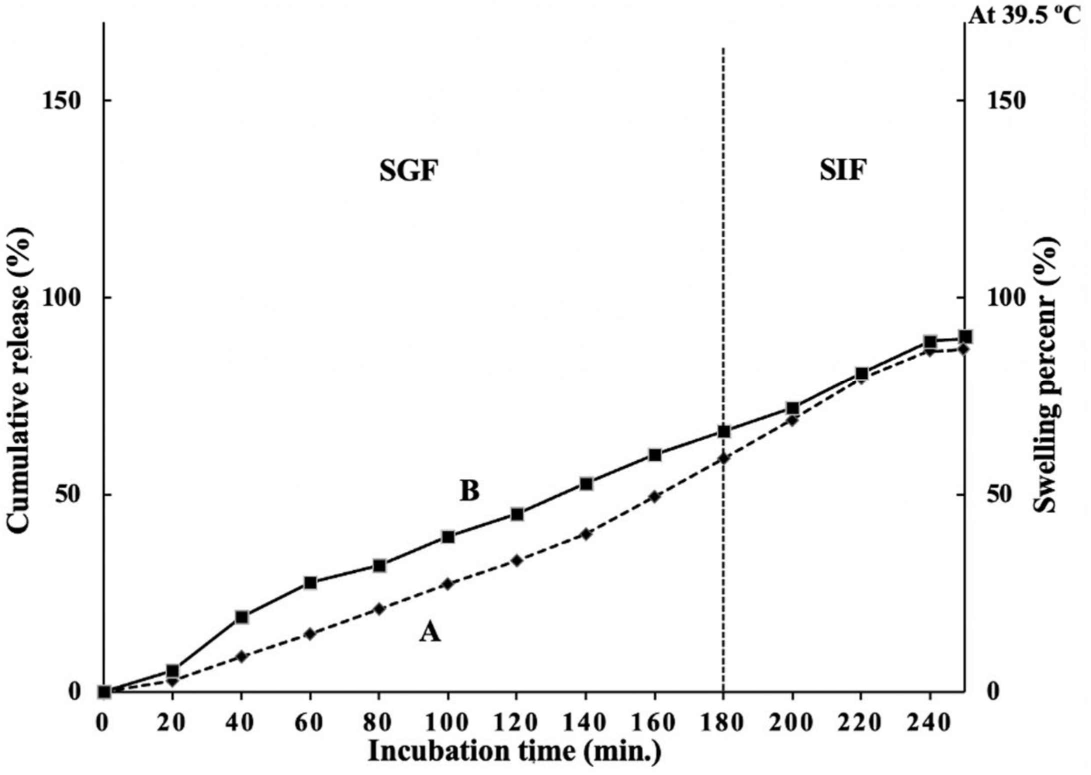

Figure S3. Performances of LPMB001/AG-AL in terms of (A) swelling percent (%), and (B) cumulative release (%) in SGF and SIF at 39.5 ± 0.5 °C.
